# Stabilization of oxidized Cu species via CeO*_x_* nano-islands for enhanced CO_2_ reduction to C_2+_ products

**DOI:** 10.1093/nsr/nwaf351

**Published:** 2025-08-23

**Authors:** Miaojin Wei, Jiawei Li, Jiankang Zhao, Sunpei Hu, Yuan Ji, Weiqing Xue, Yizhou Dai, Haoyuan Wang, Xinyan Zhang, Kwun Nam Hui, Xu Li, Chuan Xia, Tingting Zheng, Jie Zeng

**Affiliations:** Hefei National Research Center for Physical Sciences at the Microscale, Key Laboratory of Strongly-Coupled Quantum Matter Physics of Chinese Academy of Sciences, Key Laboratory of Surface and Interface Chemistry and Energy Catalysis of Anhui Higher Education Institutes, Department of Chemical Physics, University of Science and Technology of China, Hefei 230026, China; Hefei National Research Center for Physical Sciences at the Microscale, Key Laboratory of Strongly-Coupled Quantum Matter Physics of Chinese Academy of Sciences, Key Laboratory of Surface and Interface Chemistry and Energy Catalysis of Anhui Higher Education Institutes, Department of Chemical Physics, University of Science and Technology of China, Hefei 230026, China; School of Materials and Energy, University of Electronic Science and Technology of China, Chengdu 611731, China; Hefei National Research Center for Physical Sciences at the Microscale, Key Laboratory of Strongly-Coupled Quantum Matter Physics of Chinese Academy of Sciences, Key Laboratory of Surface and Interface Chemistry and Energy Catalysis of Anhui Higher Education Institutes, Department of Chemical Physics, University of Science and Technology of China, Hefei 230026, China; Hefei National Research Center for Physical Sciences at the Microscale, Key Laboratory of Strongly-Coupled Quantum Matter Physics of Chinese Academy of Sciences, Key Laboratory of Surface and Interface Chemistry and Energy Catalysis of Anhui Higher Education Institutes, Department of Chemical Physics, University of Science and Technology of China, Hefei 230026, China; School of Materials and Energy, University of Electronic Science and Technology of China, Chengdu 611731, China; Hefei National Research Center for Physical Sciences at the Microscale, Key Laboratory of Strongly-Coupled Quantum Matter Physics of Chinese Academy of Sciences, Key Laboratory of Surface and Interface Chemistry and Energy Catalysis of Anhui Higher Education Institutes, Department of Chemical Physics, University of Science and Technology of China, Hefei 230026, China; Hefei National Research Center for Physical Sciences at the Microscale, Key Laboratory of Strongly-Coupled Quantum Matter Physics of Chinese Academy of Sciences, Key Laboratory of Surface and Interface Chemistry and Energy Catalysis of Anhui Higher Education Institutes, Department of Chemical Physics, University of Science and Technology of China, Hefei 230026, China; Hefei National Research Center for Physical Sciences at the Microscale, Key Laboratory of Strongly-Coupled Quantum Matter Physics of Chinese Academy of Sciences, Key Laboratory of Surface and Interface Chemistry and Energy Catalysis of Anhui Higher Education Institutes, Department of Chemical Physics, University of Science and Technology of China, Hefei 230026, China; Hefei National Research Center for Physical Sciences at the Microscale, Key Laboratory of Strongly-Coupled Quantum Matter Physics of Chinese Academy of Sciences, Key Laboratory of Surface and Interface Chemistry and Energy Catalysis of Anhui Higher Education Institutes, Department of Chemical Physics, University of Science and Technology of China, Hefei 230026, China; Joint Key Laboratory of the Ministry of Education, Institute of Applied Physics and Materials Engineering, University of Macau Macau SAR 999078, China; School of Materials and Energy, University of Electronic Science and Technology of China, Chengdu 611731, China; School of Materials and Energy, University of Electronic Science and Technology of China, Chengdu 611731, China; School of Materials and Energy, University of Electronic Science and Technology of China, Chengdu 611731, China; Hefei National Research Center for Physical Sciences at the Microscale, Key Laboratory of Strongly-Coupled Quantum Matter Physics of Chinese Academy of Sciences, Key Laboratory of Surface and Interface Chemistry and Energy Catalysis of Anhui Higher Education Institutes, Department of Chemical Physics, University of Science and Technology of China, Hefei 230026, China; School of Chemistry & Chemical Engineering, Anhui University of Technology, Ma'anshan 243002, China

**Keywords:** CO_2_ reduction reaction, C_2+_ products, oxidized copper stabilization, CeO*_x_* nano-islands, C–C coupling efficiency

## Abstract

The presence of oxidized copper species (CuO*_x_*) on metallic Cu surfaces is widely acknowledged as a critical factor for promoting C–C coupling during the CO_2_ reduction reactions (CO_2_RR). However, the inherent instability of CuO*_x_* under negative potentials, where it is prone to reduction to metallic Cu, remains a formidable challenge. In this study, we developed a CeO*_x_*-modified CuO catalyst for the CO_2_RR, featuring CeO*_x_* uniformly distributed as isolated nano-islands on CuO nanoparticles. Upon reduction of the CuO matrix to metallic Cu, the CeO*_x_* layer effectively stabilizes the interfacial CuO*_x_*, preventing its further reduction. *Operando* characterization verified the sustained presence of Cu^2+^ and Cu^+^ species at highly reductive potentials, underscoring the role of CeO*_x_* in preserving CuO*_x_* stability. Theoretical calculations revealed that Ce^3+^ enhances the formation energy of oxygen vacancies, stabilizing the CuO*_x_* interface and *OC–CO intermediates, which are crucial for C–C coupling. With this surface modification strategy, the catalyst achieved a remarkable C_2+_ faradaic efficiency of 78% at −700 mA cm^−2^, while demonstrating persistent performance with a faradaic efficiency exceeding 70% for C_2+_ products at −100 mA cm^−2^ for over 110 h. These findings present an effective strategy for stabilizing metal oxides and advancing durable CO_2_RR catalysts.

## Teaser

CeO*_x_* nano-islands stabilize oxidized copper species under reductive conditions, enabling efficient CO_2_ reduction to multi-carbon products with 78% faradaic efficiency and 110-hour stability.

## INTRODUCTION

The rising concentration of CO_2_ in the atmosphere has led to the search for effective strategies to utilize this abundant greenhouse gas [1]. The CO_2_ reduction reaction (CO_2_RR), driven by renewable energy, offers a promising pathway for converting CO_2_ into valuable chemical products, enabling a closed-loop carbon economy [2]. Among the diverse products, C_2+_ compounds such as ethylene, ethanol and acetate are particularly attractive due to their higher economic value compared to C_1_ products [3–5]. Copper (Cu)-based catalysts are widely regarded as the most effective for facilitating C_2+_ product formation in the CO_2_RR, which is attributed to their moderate adsorption of key intermediates [6]. However, achieving high selectivity and activity for C_2+_ products remains challenging and requires precise manipulation of the reaction pathways and catalyst properties.

The efficiency of C_2+_ production is closely linked to the C–C coupling, where *CO and *CO (or *CHO) intermediates interact to form *C_2_ species [7]. One effective strategy to improve C–C coupling is to increase the surface coverage of *CO and other key intermediates [8–14]. It has been observed that Cu in higher oxidation states, such as Cu^+^/Cu^2+^, exhibits stronger CO adsorption and lower activation barriers for C–C coupling than metallic Cu does, making it a promising candidate for promoting higher-order carbon product formation [15–20]. As such, stabilizing CuO*_x_* species on Cu surfaces is essential for improving C_2+_ faradaic efficiency (FE) [21,22]. However, maintaining CuO*_x_* at the reductive potential during the CO_2_RR is challenging, as CuO*_x_* is prone to reduction to metallic Cu under reducing conditions [23–25].

Efforts to stabilize CuO*_x_* species have led to the development of several strategies [26,27]. One approach involves the synthesis of copper salts, such as Cu_3_(PO_4_)_2_, which are relatively stable and resistant to reduction [28]. While this method has yielded a high C_2+_ FE of ∼90%, the stability remains limited due to the gradual reduction of Cu^2+^ to Cu^0^, with operational lifetimes of less than 20 h. Another promising strategy is the modification of Cu with transition metal oxides (MO*_x_*), which can effectively preserve CuO*_x_* species and promote efficient C–C coupling at the CuO*_x_*/Cu interface [29–33]. Studies have explored the dispersion of Cu onto bulk MO*_x_* supports or the uniform mixing of Cu and MO*_x_* nanoparticles to maximize the interface density [34–39]. However, these configurations often compromise conductivity due to the spatial separation of Cu particles, which hinders electron transport and reduces catalytic efficiency. Therefore, achieving an optimal Cu–MO*_x_* configuration requires delicate design to balance conductivity and stability, thereby increasing C_2+_ efficiency [40–42].

Here, we present a CeO*_x_*-modified Cu catalyst (CeO*_x_*/CuO), where CeO*_x_* was dispersed as nano-islands on the Cu surface, enabling efficient CO_2_ reduction to C_2+_ products. *Operando* studies revealed that under a negative potential, bulk CuO was completely reduced to metallic Cu, whereas Cu modified with CeO*_x_* nano-islands retained its oxidation state. The CeO*_x_*/CuO catalyst, with its optimized CuO*_x_*/Cu interface, exhibited a high C_2+_ FE of 78% at a partial current density of −545 mA cm^−2^. Additionally, the CeO*_x_*/CuO catalyst demonstrated high stability, maintaining over 70% C_2+_ FE at a current density of −100 mA cm^−2^ for 110 h. Theoretical calculations supported the proposed mechanism, showing that CeO*_x_* facilitates the preservation of CuO*_x_* species, which in turn stabilize *OC–CO intermediates and enhance C–C coupling by lowering the reaction barriers.

## RESULTS AND DISCUSSION

**Figure 1.**
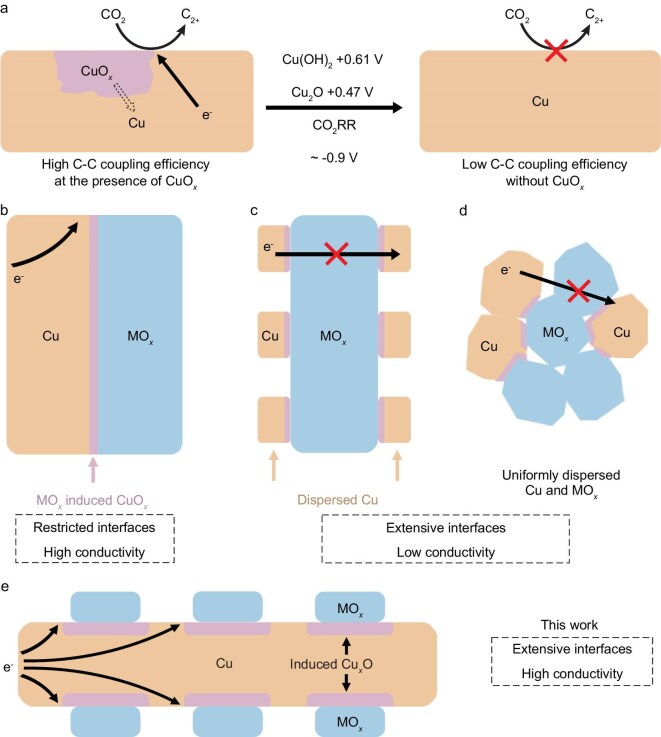
(a) CuO*_x_* (purple area) promotes the formation of C_2+_ products, but it is easily reduced to metallic Cu and thus decreases C_2+_ production. (b) CuO*_x_* forms at the interface of Cu and MO*_x_*. However, the interface between bulk MO*_x_* and bulk Cu is restricted. When Cu is dispersed on (c) bulk MO*_x_* and (d) dispersed MO*_x_*, the interface is extensive, but these assemblies suffer from the poor conductivity of bulk MO*_x_*. (e) MO*_x_* is reversely dispersed on Cu, which optimizes the balance between the interface density and conductivity.

Stabilizing CuO*_x_* under reductive conditions of the CO_2_RR is difficult because of the thermodynamic tendency of oxidized copper species to reduce to metallic Cu. As shown in Fig. 1a, the standard reduction potentials of Cu(OH)_2_ to Cu [+0.61 V vs the reversible hydrogen electrode (RHE)] and Cu_2_O to Cu (+0.47 V vs RHE) reveal their strong propensity for reduction at typical CO_2_RR operating potentials (∼−0.9 V vs RHE). This reduction undermines the stability of high-valence-state Cu, which is critical for facilitating C_2+_ product formation. To address this limitation, efforts have been directed toward stabilizing CuO*_x_* species by incorporating MO*_x_* during the CO_2_RR. Figure 1b illustrates that CuO*_x_* forms at the interface between bulk Cu and bulk MO*_x_*, but the interfacial area between Cu and MO*_x_* is limited. One approach to increase the interfacial area, as shown in Fig. 1c, involves dispersing Cu particles onto bulk MO*_x_* supports. However, this configuration often suffers from poor conductivity due to the insulating nature of bulk MO*_x_*. An alternative strategy, depicted in Fig. 1d, employs a uniform mixture of Cu and MO*_x_* nanoparticles to maximize the interface density. While this design enhances the interfacial area, it compromises the conductivity because of the spatial separation of the Cu particles. To overcome the trade-off between the interface density and conductivity, a more effective strategy is proposed in Fig. 1e, where ultra-small MO*_x_* nanoparticles are uniformly dispersed onto bulk Cu. This configuration ensures a high density of active interfaces while maintaining good conductivity, ultimately enhancing both the catalytic activity and stability. In addition to optimizing the Cu–MO*_x_* interface, selecting a metal oxide with low solubility and reduced reducibility is essential for achieving long-term stability under the reductive conditions of the CO_2_RR. Ce oxides were chosen for this study because of their inherently low solubility and reduced reducibility compared with those of other MO*_x_*, making them ideal candidates for stabilizing CuO*_x_* species.

**Figure 2.**
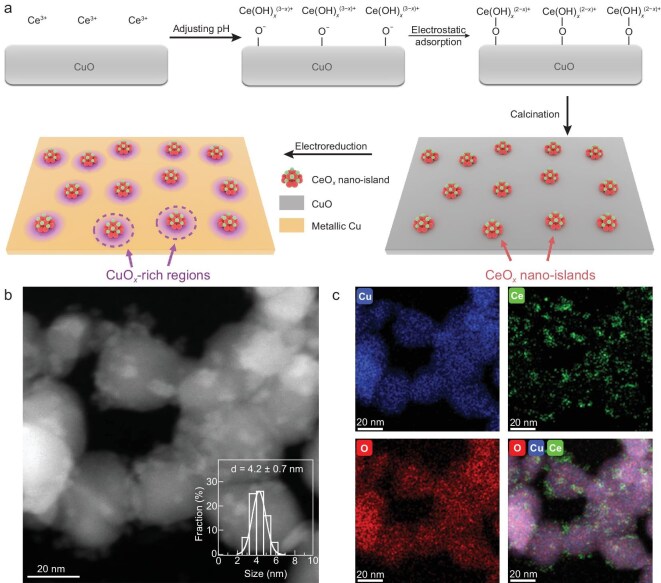
(a) Schematic illustration of the electrostatic absorption method and the CuO*_x_*-rich region introduced by CeO_2_ nano-islands after *in situ* electrochemical reduction. (b) HAADF-STEM image of CeO*_x_*/CuO. (c) The corresponding EDS mappings.

Following the identification of the optimal configuration, we explored the synthesis of Cu catalysts modified with CeO*_x_* species. The CeO*_x_*/CuO catalyst was synthesized using a strong electrostatic adsorption method (Fig. 2a) [43]. First, CuO nanoparticles were synthesized via the calcination of CuC_2_O_4_ at 623 K in air, and then 20–50 nm CuO nanoparticles were obtained (Fig. S1). For the deposition of CeO*_x_*, Ce(NO_3_)_3_ was dissolved in a suspension of CuO nanoparticles, and the pH of the solution was adjusted by adding KOH to exceed the point of zero charge (pzc ∼7.6) of CuO while remaining below the critical pH where Ce^3+^ precipitates as Ce(OH)_3_ (solubility product constant K_sp_ = 1.6 × 10^−20^). This resulted in the formation of Ce(OH)*_x_*^+^ clusters, which were adsorbed onto the negatively charged CuO surface. Instead of aggregating, these clusters formed separated nano-islands due to electrostatic repulsion. The loading of CeO*_x_* nano-islands could be controlled by adjusting the amount of KOH added. After calcination in air at 623 K, the CeO*_x_*/CuO catalyst was successfully synthesized.

We then characterized the as-synthesized catalysts using various techniques. Inductively coupled plasma optical emission spectrometry (ICP-OES) analysis revealed a Ce/Cu mass ratio of 0.02 (Table S1). X-ray diffraction (XRD) patterns (Fig. S2) revealed predominant peaks for monoclinic CuO (space group *C*2/*c*) in both CeO*_x_*/CuO and CuO, along with minor peaks for cubic CeO_2_ (space group *Fm*-3*m*) in the CeO*_x_*/CuO catalysts. High-angle annular dark-field scanning transmission electron microscopy (HAADF-STEM) images (Fig. 2b) revealed the presence of numerous nano-islands with an average diameter of 4.2 nm uniformly distributed on the CuO surface. These nano-islands exhibited a consistent morphology across a range of transmission electron microscopy (TEM) images (Fig. S3). Energy dispersive spectroscopy (EDS) elemental mapping (Fig. 2c) confirmed that the nano-islands consisted of Ce (green) and O (red). High-resolution TEM (HRTEM) images of CeO*_x_*/CuO revealed distinct lattice fringes for CeO_2_ and CuO, with spacings of 0.32 and 0.28 nm for the CeO_2_ (111) and (200) planes, respectively, and 0.18 nm for the CuO (112) plane (Fig. S4). The Ce 3*d* X-ray photoelectron spectroscopy (XPS) revealed 26.1% Ce^3+^ species in CeO*_x_*/CuO (*x* = 1.87, Fig. S5a), indicating the existence of oxygen vacancies in the ceria phase. The Cu *LM*2 Auger spectra of CeO*_x_*/CuO suggested that Cu was predominantly in the oxidation state of +2 (Fig. S5b). These results confirmed the presence of approximately 4 nm CeO*_x_* particles dispersed on CuO. To investigate the adaptability of the electrostatic adsorption method, we varied the Ce loading by adjusting the amount of KOH added during synthesis. As shown in Fig. S6, increasing the KOH beyond the optimal point (∼2% Ce) did not lead to additional Ce incorporation onto the CuO surface. Instead, it resulted in the formation of discrete Ce-containing precipitates, indicating saturation of available adsorption sites. Conversely, reducing the Ce precursor led to catalysts with lower Ce loadings, which were systematically characterized by ICP-OES (Table S1), and electron microscopy (Fig. S7).

**Figure 3.**
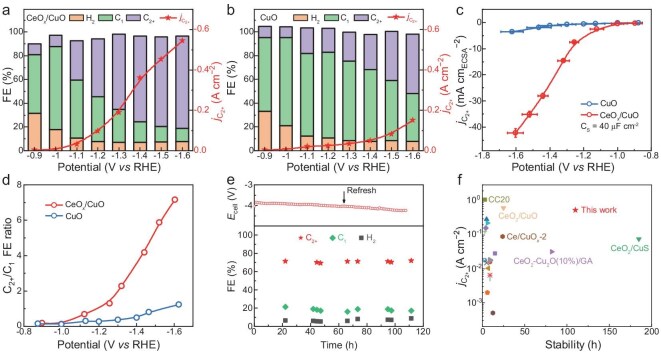
Distribution of products in the CO_2_RR and partial current densities of the C_2+_ product at different potentials in a flow cell of the (a) CeO*_x_*/CuO and (b) CuO catalysts. (c) Variation in the ECSA-correlated partial current density of the C_2+_ product against the applied potential over CeO*_x_*/CuO (red) and CuO (blue). (d) Ratio of C_2+_ to C_1_ products at different potentials. (e) Duration test of CeO*_x_*/CuO in an MEA reactor. (f) Comparison of the partial current densities of C_2+_ products and long-term stabilities of CeO*_x_*/CuO with those of other CuCe-based catalysts reported in the literature.

To evaluate the C–C coupling efficiencies of the CeO*_x_*/CuO and CuO catalysts, the CO_2_RR was conducted in a flow-cell configuration. Across all applied potentials, the CeO*_x_*/CuO catalyst consistently exhibited a higher C_2+_ FE than CuO did (Fig. 3a and b; detailed product distribution in Fig. S8a and b, with error bars of at least four independent tests). Notably, the CeO*_x_*/CuO catalyst achieved a maximum C_2+_ FE of 78% with a partial current density of −545 mA cm^−2^ at −1.6 V vs RHE, with ethylene (C_2_H_4_) as the primary product (48% FE, Fig. S8a). In contrast, the CuO catalyst without CeO*_x_* modification produced CO as the main product (Fig. S8b). As can be seen, the deposition of CeO*_x_* on the CuO catalyst significantly enhanced both the C_2+_ FEs and partial current densities of the C_2+_ products (Fig. 3a and b). To investigate whether the increased current density resulted from a greater active surface area, we measured the double-layer capacitance to determine the electrochemically active surface area (ECSA). Surprisingly, the ECSA of CeO*_x_*/CuO was smaller than that of CuO (Fig. S9), despite the similar CuO morphologies of both catalysts. This reduction in the ECSA is likely due to the increased hydrophobicity introduced by surface modification with CeO*_x_* nano-islands [44]. The water contact angle of CuO is 19.4° (Fig. S10a), while the contact angle increased to 66.6° after CeO*_x_* nano-islands were introduced (Fig. S10b). Based on the ECSA measurements, the ECSA-normalized partial current density for the C_2+_ products was calculated against the applied potential for both catalysts (Fig. 3c). The results revealed significantly higher normalized C_2+_ current densities for CeO*_x_*/CuO, confirming its superior intrinsic activity in the CO_2_RR. We also conducted the CO_2_RR under both acidic and alkaline conditions (Fig. S11). At a current density of −600 mA cm^−2^, the CeO*_x_*/CuO catalyst consistently exhibited over 70% C_2+_ FE, indicating that CeO*_x_* nano-islands effectively promote C_2+_ generation across diverse environments, and also demonstrating the broad applicability of this catalyst.

**Figure 4.**
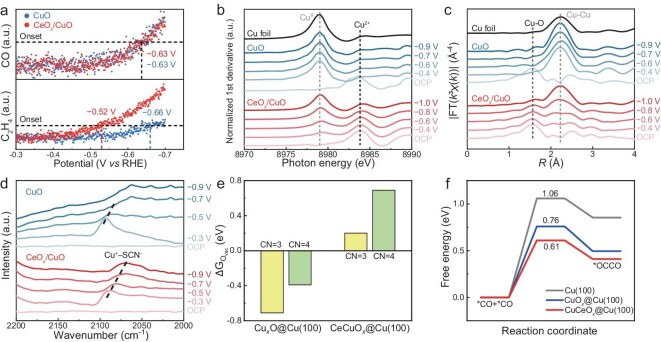
(a) *Operando* DEMS of CO and C_2_H_4_ during the CO_2_RR on the CeO*_x_*/CuO and CuO catalysts. (b) First derivatives of the *operando* Cu K-edge XANES spectra of CuO and CeO*_x_*/CuO at different potentials. (c) *Operando* EXAFS spectra of the CuO and CeO*_x_*/CuO catalysts at different potentials. (d) *Operando* ATR-SEIRAS of CuO and CeO*_x_*/CuO in CO_2_-purged 0.1 M KHCO_3_ at different potentials, with 0.04 M KSCN used as a probe molecule for the generation of Cu^+^ during electrolysis. (e) Oxygen vacancy formation energies of different coordination numbers on different surfaces. (f) Reaction barriers of *CO dimerization on various surfaces.

To further examine the C–C coupling efficiency, the C_2+_/C_1_ product FE ratio was calculated for both catalysts (Fig. 3d). Compared with the CuO catalyst, the CeO*_x_*/CuO catalyst presented a significantly higher C_2+_/C_1_ ratio. At −1.4 V vs RHE, the CeO*_x_*/CuO catalyst had a C_2+_/C_1_ ratio of 4.2, which was seven times greater than that of the CuO catalyst (a C_2+_/C_1_ ratio of 0.5). At −1.6 V vs RHE, the C_2+_/C_1_ ratio of the CeO*_x_*/CuO catalyst reached as high as 7.2, whereas that of the CuO catalyst remained at 1.2, indicating that CeO*_x_* nano-island modification enhanced the C–C coupling efficiency. The CeO*_x_*/CuO catalyst also demonstrated high stability in the CO_2_RR. In a flow cell configuration, the CeO*_x_*/CuO catalyst exhibited >40% C_2_H_4_ FE at a high current density of −400 mA cm^−2^ for more than 15 h (Fig. S12a). The performance dropped after 15 h due to salt precipitate and flooding (Fig. S12b and c). To evaluate the intrinsic stability of the CeO*_x_*/CuO catalyst under industrially relevant conditions, a duration test was also conducted in a membrane electrode assembly (MEA) configuration (Fig. 3e). The CeO*_x_*/CuO catalyst maintained a C_2+_ FE exceeding 70% at a current density of −100 mA cm^−2^ for over 110 h. Post-catalysis analysis confirmed the retention of the CeO*_x_* structure. High-resolution TEM images verified the dispersion of CeO*_x_* nano-islands across the Cu surface (Fig. S13). The *quasi-in situ* Ce 3*d* XPS spectra revealed 35.0% Ce^3+^ species on CeO*_x_*/CuO (*x* = 1.83), which indicated that the CeO*_x_*/CuO catalyst was stable because of its low solubility and reduced reducibility of CeO*_x_* (Fig. S5c). The XRD pattern of CeO*_x_*/CuO after reaction showed a higher Cu_2_O(111)/Cu(111) peak ratio than that of CuO (Fig. S14). These results demonstrated the structural stability of the catalysts throughout the CO_2_RR process. Compared with other CeCu-based catalysts reported in previous studies [29–31,34,39–42,45–55] (Fig. 3f and Fig. S15), the CeO*_x_*/CuO catalyst outperformed these materials, demonstrating both a high partial current density for C_2+_ products and robust stability. The improved performance was presumably attributed to the inertness of CeO*_x_* and the hydrophobicity introduced by the nano-islands, which together enhance the stability and activity of the catalyst. Unexpectedly, the productivity of CO, a representative C_1_ product, was not suppressed in the presence of CeO*_x_* nano-islands, as demonstrated by *operando* differential electrochemical mass spectrometry (DEMS, Fig. 4a). Both the CeO*_x_*/CuO and CuO catalysts exhibited similar onset potentials (−0.63 V vs RHE) for CO production. However, the onset potential for C_2_H_4_ on CeO*_x_*/CuO was −0.52 V vs RHE, which is significantly more positive than that on CuO (−0.66 V vs RHE), indicating that the *CO coverage required for effective C–C coupling on CeO*_x_*/CuO was lower. This observation suggested that the CeO*_x_* nano-islands enhanced the C–C coupling process without inhibiting C_1_ production.

To investigate the impact of CeO*_x_* nano-islands on the electronic structure of Cu during the CO_2_RR, *operando* X-ray absorption near edge structure (XANES) and extended X-ray absorption fine structure (EXAFS) analyses were performed. The first derivatives of the XANES data (Fig. 4b) revealed that CuO was quickly reduced to metallic Cu under negative potentials. In contrast, CeO*_x_*/CuO exhibited features of both metallic Cu and Cu^2+^, indicating partial reduction. The Cu^2+^ content decreased progressively with increasing negative potential, but did not fully disappear, suggesting that the CeO*_x_* nano-islands effectively stabilized Cu^2+^. The white-line peaks of CeO*_x_*/CuO (Fig. S16a) displayed characteristics of both Cu^2+^ and metallic Cu, whereas CuO (Fig. S16b) showed only metallic Cu features after reduction. These results highlight that CeO*_x_* nano-islands impede the full reduction of Cu, preserving a fraction of Cu in its higher oxidation state. The EXAFS spectra further supported these findings. For CuO (Fig. 4c), the Cu–O bonds disappeared at modestly negative potentials, whereas the Cu–Cu bonds formed immediately upon the application of a negative potential, indicating that CuO was highly susceptible to reduction. In contrast, for CeO*_x_*/CuO, the number of Cu–O bonds gradually decreased as the potential became more negative, persisting even under highly reductive conditions. Cu–Cu bonds only became dominant at −0.8 V vs RHE or at more negative potentials, confirming that the presence of CeO*_x_* nano-islands hindered the reduction of CuO.

*Operando* Raman spectroscopy provided further evidence supporting the stabilizing effect of CeO*_x_* nano-islands on Cu–O species (Fig. S17). Peaks at approximately 600 cm^−1^, attributed to surface Cu–O species, disappeared on the CuO catalyst under negative potentials but remained detectable on the CeO*_x_*/CuO catalyst, confirming the ability of CeO*_x_* nano-islands to preserve surface Cu–O species under reducing conditions. Peaks at 800 cm^−1^ were attributed to peroxide vibration on CeO*_x_*(111) [56]. This peak is absent in the CuO sample, confirming the successful deposition of CeO*_x_* in the CeO*_x_*/CuO composite. To directly probe the presence of surface Cu oxidized species on CeO*_x_*/CuO, *operando* attenuated total reflection surface-enhanced infrared absorption spectroscopy (ATR-SEIRAS) was conducted using SCN^−^ as a probe molecule [57]. As shown in Fig. 4d, the peak at approximately 2073 cm^−1^ was attributed to Cu^+^-SCN^−^ bonding. On the CuO surface, Cu^+^ initially formed as the potential became negative, increasing in intensity before diminishing and disappearing below the detection limit at potentials more negative than −0.7 V vs RHE. In contrast, on the CeO*_x_*/CuO surface, the Cu^+^–SCN^−^ peaks followed a similar trend of initial increase and subsequent decrease but remained detectable throughout the entire test. These results indicate that Cu^+^ species formed on both catalysts; however, the Cu^+^ on the CuO catalyst was more readily reduced to metallic Cu, whereas the Cu^+^ on CeO*_x_*/CuO was stabilized by the CeO*_x_* nano-islands, persisting even under highly reductive conditions. Furthermore, the *quasi-in situ* Cu *LM*2 Auger spectra of CeO*_x_*/CuO after the duration test revealed a mixture of metallic Cu, Cu^+^ and Cu^2+^ features (Fig. S5d), indicating that the higher oxidation state of Cu was stabilized by CeO*_x_*. This highlights the critical role of CeO*_x_* nano-islands in maintaining the active Cu^+^ species, which are essential for efficient CO_2_RR and enhanced C–C coupling.

Based on these characterizations, we propose a model in which CeO*_x_* nano-islands create and stabilize CuO*_x_* regions on the CeO*_x_*/CuO catalyst during the CO_2_RR (Fig. 2a, highlighted in purple circles). The CeO*_x_* nano-islands effectively modulate the electronic structure of Cu, allowing the retention of Cu in oxidized states under reducing conditions. These CuO*_x_* regions serve as critical active sites, facilitating C–C coupling and thereby significantly enhancing the C_2+_ efficiency. In addition, the inert nature of CeO*_x_* and the hydrophobicity introduced by the nano-islands also contributed to the stabilization of the catalyst system, enabling sustained performance during long-term tests. This combination of activity enhancement and stability improvement highlights the effectiveness of CeO*_x_* nano-islands in optimizing the performance of the CeO*_x_*/CuO catalyst.

To delve into the origin of CeO*_x_* nano-islands enhancing C–C coupling activity on Cu, we further conducted theoretical calculations. We constructed Cu_6_O_6_ and Ce_3_Cu_3_O_9_ clusters on Cu (100) to compare the differences in oxygen vacancy (O_v_) formation energy and the energy barriers of CO dimerization among the Cu (100), CuO*_x_*–Cu (100) and CeCuO*_x_*–Cu (100) surfaces or interfaces. For CuO*_x_* species on a Cu surface, the Cu–O–Cu structure readily forms oxygen vacancies, indicating that Cu is prone to reduction (Fig. 4e). The free energies for forming vacancies at oxygen sites coordinated by three Cu atoms (Fig. S18a, yellow circle) and four Cu atoms (Fig. S18a, green circle) are −0.71 and −0.39 eV, respectively. In contrast, introducing CeO*_x_* clusters results in the formation of Ce–O–Cu structures, where oxygen atoms are less susceptible to reduction. The vacancy formation free energies at analogous sites are +0.20 (Fig. S18b, yellow circle) and +0.69 eV (Fig. S18b, green circle). This suggests that CeO*_x_* clusters help retain more oxygen on the Cu surface, thereby increasing the surface Cu valence state, which, as previously discussed, promotes C–C coupling. We further investigated the mechanism by which CuO*_x_* species enhance C–C coupling. In the Cu-based CO_2_RR, multiple intermediates are involved in C–C coupling. To explore this process, we selected *CO dimerization as a model to compare the CeO*_x_*/CuO catalyst with metallic Cu. On the Cu (100) surface, the coupling of bridge-adsorbed *CO + *CO involves four Cu atoms (Fig. S19a) and requires a high activation energy of 1.06 eV (Fig. 4f), indicating that C–C coupling is challenging on a metallic Cu surface. However, in the presence of CuO*_x_*, the oxidized Cu stabilizes the *OC–CO intermediate through interactions with the oxygen atom in *CO (Fig. S19b), lowering the activation energy to 0.76 eV (Fig. 4f). Furthermore, Ce^3+^ ions in CeO*_x_* clusters also stabilize the *OC–CO intermediate (Fig. S19c), further reducing the energy barrier for *CO + *CO coupling to 0.61 eV on the CeCuO*_x_*@Cu (100) surface (Fig. 4f). This significant reduction in the energy barrier demonstrated that CeO*_x_* nano-islands and CuO*_x_* species synergistically facilitated C–C coupling. These findings explain how the synergistic interaction between CeO*_x_* nano-islands and CuO*_x_* species enhances C–C coupling activity during the CO_2_RR. The Ce–O–Cu structures at the CeO*_x_–*CuO*_x_* interface enhance the oxygen stability and maintain higher Cu valence states, whereas the stabilization of *OC–CO intermediates by CeO*_x_* and CuO*_x_* promotes efficient C_2+_ product formation in the CO_2_RR.

Enhancing the FE of the target product holds significant importance for reducing the cost of electrochemical production. We analyzed the differences between CeO*_x_*/CuO and CuO catalysts for ethylene production from these three perspectives (Fig. S20, Supplementary Note 1). Following the substitution of CuO with CeO*_x_*/CuO for ethylene generation, carbon capture costs decreased by 70.2%, electrolysis energy costs decreased by 29.2% and ethylene separation costs decreased by 62.7%. These results demonstrate the promising potential of the CeO*_x_*/CuO catalyst for industrial applications.

## CONCLUSION

In summary, we demonstrated an effective strategy for constructing CeO*_x_* nano-islands on Cu particles to promote C–C coupling efficiency in the CO_2_RR. *Operando* XAFS and ATR-SEIRAS combined with *quasi-in situ* XPS revealed that CeO*_x_* nano-islands effectively stabilize Cu^+^ and Cu^2+^ species under reductive conditions, preserving the active oxidation states necessary for sustained catalytic performance. The catalyst achieved a remarkable FE of 78% for C_2+_ products at −700 mA cm^−2^, with durability maintaining over 70% FE at −100 mA cm^−2^ for more than 110 h. Theoretical calculations revealed that CeO*_x_* promotes CuO*_x_* formation, which is crucial for C_2+_ production, lowering the energy barrier for C–C coupling by stabilizing *OC–CO intermediates. This study underscores the dual role of CeO*_x_* as both a stabilizer of CuO*_x_* and a promoter of C_2+_ production. Importantly, this study highlights the potential for further optimization of activity, selectivity and stability by exploring alternative elements as modifiers, paving the way for scalable and economically viable CO_2_RR technologies.

## Supplementary Material

nwaf351_Supplemental_File

## Data Availability

All data are available in the main text or the Supplementary data.

## References

[1] Hepburn C, Adlen E, Beddington J et al. The technological and economic prospects for CO_2_ utilization and removal. Nature 2019; 575: 87–97. 10.1038/s41586-019-1681-6 31695213

[2] De Luna P, Hahn C, Higgins D et al. What would it take for renewably powered electrosynthesis to displace petrochemical processes? Science 2019; 364: eaav3506. 10.1126/science.aav3506 31023896

[3] Dinh C-T, Burdyny T, Kibria MG et al. CO_2_ electroreduction to ethylene via hydroxide-mediated copper catalysis at an abrupt interface. Science 2018; 360: 783–7. 10.1126/science.aas9100 29773749

[4] Ding J, Yang HB, Ma X-L et al. A tin-based tandem electrocatalyst for CO_2_ reduction to ethanol with 80% selectivity. Nat Energy 2023; 8: 1386–94. 10.1038/s41560-023-01389-3

[5] Yuan L-J, Sui X-L, Pan H et al. Strategies and mechanism for enhancing intrinsic activity of metal–nitrogen–carbon catalysts in electrocatalytic reactions. Renewables 2023; 1: 514–40. 10.31635/renewables.023.202300023

[6] Gao D, Arán-Ais RM, Jeon HS et al. Rational catalyst and electrolyte design for CO_2_ electroreduction towards multicarbon products. Nat Catal 2019; 2: 198–210. 10.1038/s41929-019-0235-5

[7] Wang Y, Liu J, Zheng G. Designing copper-based catalysts for efficient carbon dioxide electroreduction. Adv Mater 2021; 33: e2005798. 10.1002/adma.202005798 33913569

[8] Li J, Chang X, Zhang H et al. Electrokinetic and in situ spectroscopic investigations of CO electrochemical reduction on copper. Nat Commun 2021; 12: 3264. 10.1038/s41467-021-23582-2 34075039 PMC8169934

[9] Deng W, Zhang P, Qiao Y et al. Unraveling the rate-determining step of C_2+_ products during electrochemical CO reduction. Nat Commun 2024; 15: 892. 10.1038/s41467-024-45230-1 38291057 PMC10828390

[10] Zhan C, Dattila F, Rettenmaier C et al. Key intermediates and Cu active sites for CO_2_ electroreduction to ethylene and ethanol. Nat Energy 2024; 9: 1485–96. 10.1038/s41560-024-01633-4 39713047 PMC11659170

[11] Bian L, Bai Y, Chen J-Y et al. Hierarchical tandem catalysis promotes CO spillover and trapping for efficient CO_2_ reduction to C_2+_ products. ACS Nano 2025; 19: 9304–16. 10.1021/acsnano.5c00696 40016093

[12] Chen L, Chen J, Fu W et al. Energy-efficient CO_(2)_ conversion to multicarbon products at high rates on CuGa bimetallic catalyst. Nat Commun 2024; 15: 7053. 10.1038/s41467-024-51466-8 39147764 PMC11327302

[13] Zhao Y, Liu X, Chen J et al. Promote electroreduction of CO_2_ via catalyst valence state manipulation by surface-capping ligand. Proc Natl Acad Sci USA 2023; 120: e2218040120. 10.1073/pnas.2218040120 37216512 PMC10235936

[14] Fu W, Li Y, Chen J et al. Preserving molecular tuning for enhanced electrocatalytic CO_2_-to-ethanol conversion. Angew Chem 2024; 136: e202407992. 10.1002/ange.202407992 39140436

[15] Li H, Jiang Y, Li X et al. C_2+_ selectivity for CO_2_ electroreduction on oxidized Cu-based catalysts. J Am Chem Soc 2023; 145: 14335–44. 10.1021/jacs.3c03022 37342888

[16] Wu H, Huang L, Timoshenko J et al. Selective and energy-efficient electrosynthesis of ethylene from CO_2_ by tuning the valence of Cu catalysts through aryl diazonium functionalization. Nat Energy 2024; 9: 422–33. 10.1038/s41560-024-01461-6

[17] Herzog A, Luna ML, Jeon HS et al. Operando Raman spectroscopy uncovers hydroxide and CO species enhance ethanol selectivity during pulsed CO_2_ electroreduction. Nat Commun 2024; 15: 3986. 10.1038/s41467-024-48052-3 38734726 PMC11088695

[18] Lin S-C, Chang C–C, Chiu S-Y et al. Operando time-resolved X-ray absorption spectroscopy reveals the chemical nature enabling highly selective CO_2_ reduction. Nat Commun 2020; 11: 3525. 10.1038/s41467-020-17231-3 32665607 PMC7360608

[19] Yang H, Li S, Xu Q. Efficient strategies for promoting the electrochemical reduction of CO_2_ to C_2+_ products over Cu-based catalysts. Chin J Catal 2023; 48: 32–65. 10.1016/S1872-2067(23)64429-8

[20] Timoshenko J, Bergmann A, Rettenmaier C et al. Steering the structure and selectivity of CO_2_ electroreduction catalysts by potential pulses. Nat Catal 2022; 5: 259–67. 10.1038/s41929-022-00760-z

[21] Cao Y, Chen Z, Li P et al. Surface hydroxide promotes CO_2_ electrolysis to ethylene in acidic conditions. Nat Commun 2023; 14: 2387. 10.1038/s41467-023-37898-8 37185342 PMC10130127

[22] Liu H, Yang C, Bian T et al. Bottom-up growth of convex sphere with adjustable Cu(0)/Cu(I) interfaces for effective C_2_ production from CO_2_ electroreduction. Angew Chem Int Ed 2024; 63: e202404123. 10.1002/anie.202404123 38702953

[23] Yang Y, Louisia S, Yu S et al. Operando studies reveal active Cu nanograins for CO_2_ electroreduction. Nature 2023; 614: 262–9. 10.1038/s41586-022-05540-0 36755171

[24] Lian Z, Dattila F, López N. Stability and lifetime of diffusion-trapped oxygen in oxide-derived copper CO_2_ reduction electrocatalysts. Nat Catal 2024; 7: 401–11. 10.1038/s41929-024-01132-5

[25] Choi W, Chae Y, Liu E et al. Exploring the influence of cell configurations on Cu catalyst reconstruction during CO_2_ electroreduction. Nat Commun 2024; 15: 8345. 10.1038/s41467-024-52692-w . 39333114 PMC11437247

[26] Feng J, Wu L, Liu S et al. Improving CO_2_‑to‑C_2+_ product electroreduction efficiency via atomic lanthanide dopant-induced tensile-strained CuO*_x_* catalysts. J Am Chem Soc 2023; 145: 9857–66. 10.1021/jacs.3c02428 37092347

[27] Du Y-R, Li X-Q, Duan G-Y et al. Sn-based redox cycle mediated microenvironment regulation of Cu sites on poly(ionic liquid) enhance electrocatalytic CO-to-C_2+_ conversion. Appl Catal B Environ 2023; 337: 122969. 10.1016/j.apcatb.2023.122969

[28] Zhang XY, Lou ZX, Chen J et al. Direct OC–CHO coupling towards highly C_2+_ products selective electroreduction over stable Cu^0^/Cu^2+^ interface. Nat Commun 2023; 14: 7681. 10.1038/s41467-023-43182-6 37996421 PMC10667242

[29] Zhao Z, Li X, Wang J et al. CeO_2_-modified Cu electrode for efficient CO_2_ electroreduction to multi-carbon products. J CO_2_ Util 2021; 54: 101741. 10.1016/j.jcou.2021.101741

[30] Wang H, Zhang H, Huang Y et al. Strain in copper/ceria heterostructure promotes electrosynthesis of multicarbon products. ACS Nano 2023; 17: 346–54. 10.1021/acsnano.2c08453 36574462

[31] Yang Z, Ji D, Li Z et al. CeO_2_/CuS nanoplates electroreduce CO_2_ to ethanol with stabilized Cu^+^ species. Small 2023; 19: e2303099. 10.1002/smll.202303099 37269214

[32] Tian H, Yang J-T, Wang X et al. Ionic liquid-TiO_2_-CuO_x_ composite interfaces combined with gas directional transmission for enhanced electrooxidation of methane to ethanol. Appl Catal B Environ 2025; 375: 125411. 10.1016/j.apcatb.2025.125411

[33] Tian H, Zhang Z-Y, Fang H et al. Selective electrooxidation of methane to formic acid by atomically dispersed CuO_x_ and its induced Lewis acid sites on V_2_O_5_ in a tubular electrode. Appl Catal B Environ 2024; 351: 124001. 10.1016/j.apcatb.2024.124001

[34] Hong S, Abbas HG, Jang K et al. Tuning the C_1_/C_2_ selectivity of electrochemical CO_2_ reduction on Cu–CeO_2_ nanorods by oxidation state control. Adv Mater 2023; 35: e2208996. 10.1002/adma.202208996 36470580

[35] Sultan S, Lee H, Park S et al. Interface rich CuO/Al_2_CuO_4_ surface for selective ethylene production from electrochemical CO_2_ conversion. Energy Environ Sci 2022; 15: 2397–409. 10.1039/D1EE03861C

[36] Hu X, Xu J, Gao Y et al. Establishing non-stoichiometric Ti_4_O_7_ assisted asymmetrical C–C coupling for highly energy-efficient electroreduction of carbon monoxide. Angew Chem Int Ed 2025; 64: e202414416. 10.1002/anie.202414416 39435844

[37] Chang C-J, Lai Y-A, Chu Y-C et al. Lewis acidic support boosts C–C coupling in the pulsed electrochemical CO_2_ reaction. J Am Chem Soc 2023; 145: 6953–65. 10.1021/jacs.3c00472 36921031

[38] Kou T, Wang S, Yang S et al. Amorphous CeO_2__–_Cu heterostructure enhances CO_2_ electroreduction to multicarbon alcohols. ACS Mater Lett 2022; 4: 1999–2008. 10.1021/acsmaterialslett.2c00506

[39] Sun Y, Xie J, Fu Z et al. Boosting CO_2_ electroreduction to C_2_H_4_ via unconventional hybridization: high-order Ce^4+^ 4f and O 2p interaction in Ce-Cu_2_O for stabilizing Cu^+^. ACS Nano 2023; 17: 13974–84. 10.1021/acsnano.3c03952 37410800

[40] Luo M, Wang Z, Li YC et al. Hydroxide promotes carbon dioxide electroreduction to ethanol on copper via tuning of adsorbed hydrogen. Nat Commun 2019; 10: 5814. 10.1038/s41467-019-13833-8 31862886 PMC6925210

[41] Varandili SB, Huang J, Oveisi E et al. Synthesis of Cu/CeO_2‑x_ nanocrystalline heterodimers with interfacial active sites to promote CO_2_ electroreduction. ACS Catal 2019; 9: 5035–46. 10.1021/acscatal.9b00010

[42] Yan X, Chen C, Wu Y et al. Efficient electroreduction of CO_2_ to C_2+_ products on CeO_2_ modified CuO. Chem Sci 2021; 12: 6638–45. 10.1039/D1SC01117K 34040738 PMC8132937

[43] Li X, Pereira-Hernandez XI, Chen Y et al. Functional CeO*_x_* nanoglues for robust atomically dispersed catalysts. Nature 2022; 611: 284–8. 10.1038/s41586-022-05251-6 36289341

[44] Azimi G, Dhiman R, Kwon HM et al. Hydrophobicity of rare-earth oxide ceramics. Nature Mater 2013; 12: 315–20. 10.1038/nmat3545 23333998

[45] Wu D, Dong C, Wu D et al. Cuprous ions embedded in ceria lattice for selective and stable electrochemical reduction of carbon dioxide to ethylene. J Mater Chem A 2018; 6: 9373–7. 10.1039/C8TA01677A

[46] Yin J, Gao Z, Wei F et al. Customizable CO_2_ electroreduction to C_1_ or C_2+_ products through Cu_y_/CeO_2_ interface engineering. ACS Catal 2022; 12: 1004–11. 10.1021/acscatal.1c04714

[47] Shan J, Shi Y, Li H et al. Effective CO_2_ electroreduction toward C_2_H_4_ boosted by Ce-doped Cu nanoparticles. Chem Eng J 2022; 433: 133769. 10.1016/j.cej.2021.133769

[48] Chu M, Chen C, Wu Y et al. Enhanced CO_2_ electroreduction to ethylene via strong metal-support interaction. Green Energy Environ 2022; 7: 792–8. 10.1016/j.gee.2020.12.001

[49] Tian Y, Fei X, Ning H et al. Membrane-free electrocatalysis of CO_2_ to C_2_ on CuO/CeO_2_ nanocomposites. Front Chem 2022; 10: 915759. 10.3389/fchem.2022.915759 35755265 PMC9215358

[50] Yap FM, Loh JY, Yuan S et al. Revolutionizing CO_2_-to-C_2_ conversion: unleashing the potential of CeO_2_ nanocores for self-supported electrocatalysts with Cu_2_O nanoflakes on 3D graphene aerogel. Adv Funct Mater 2025; 35: 2407605. 10.1002/adfm.202407605

[51] Chu S, Yan X, Choi C et al. Stabilization of Cu^+^ by tuning a CuO–CeO_2_ interface for selective electrochemical CO_2_ reduction to ethylene. Green Chem 2020; 22: 6540–6. 10.1039/D0GC02279A

[52] Tan D, Wulan B, Cao X et al. Strong interactions of metal-support for efficient reduction of carbon dioxide into ethylene. Nano Energy 2021; 89: 106460. 10.1016/j.nanoen.2021.106460

[53] Cai H-D, Nie B, Guan P et al. Tuning the interactions in CuO nanosheet-decorated CeO_2_ nanorods for controlling the electrochemical reduction of CO_2_ to methane or ethylene. ACS Appl Nano Mater 2022; 5: 7259–67. 10.1021/acsanm.2c01164

[54] Liu X, Liu T, Ouyang T et al. Ce^3+^/Ce^4+^ ion redox shuttle stabilized Cu^δ+^ for efficient CO_2_ electroreduction to C_2_H_4_. Angew Chem Int Ed 2025; 64: e202419796. 10.1002/anie.202419796 39589341

[55] Qiao Y, Shen S, Mao C et al. Interfacial oxygen vacancy-copper pair sites on inverse CeO_2_/Cu catalyst enable efficient CO_2_ electroreduction to ethanol in acid. Angew Chem Int Ed 2025; 64: e202424248. 10.1002/anie.202424248 39788905

[56] Schilling C, Hofmann A, Hess C et al. Raman spectra of polycrystalline CeO_2_: a density functional theory study. J Phys Chem C 2017; 121: 20834–49. 10.1021/acs.jpcc.7b06643

[57] Wu J, Xu L, Kong Z et al. Integrated tandem electrochemical-chemical-electrochemical coupling of biomass and nitrate to sustainable alanine. Angew Chem Int Ed 2023; 62: e202311196. 10.1002/anie.202311196 37721394

