# Supplementary material for: Stabilization of oxidized Cu species via CeOx nano-islands for enhanced CO2 reduction to C2+ products
Source: Natl Sci Rev. 2025 Aug 23;12(11):nwaf351. doi: 10.1093/nsr/nwaf351 (PMC12547411; doi:10.1093/nsr/nwaf351)
Supplement: nwaf351_Supplemental_File [file nwaf351_supplemental_file.pdf]

Supplementary Information for

Stabilization of Oxidized Cu Sites via CeO_x Nano-islands for CO_2 Reduction to C_{2+} Products

Miaojin Wei[1†], Jiawei Li[1,2†], Jiankang Zhao[1], Sunpei Hu[1], Yuan Ji[2], Weiqing Xue[1], Yizhou Dai[1], Haoyuan Wang[1], Xinyan Zhang[1], Kwun Nam Hui[3], Xu Li[2], Chuan Xia[2], Tingting Zheng[2], Jie Zeng[1,4]*

[1]Hefei National Research Center for Physical Sciences at the Microscale, Key Laboratory of Strongly-Coupled Quantum Matter Physics of Chinese Academy of Sciences, Key Laboratory of Surface and Interface Chemistry and Energy Catalysis of Anhui Higher Education Institutes, Department of Chemical Physics, University of Science and Technology of China, Hefei, Anhui 230026, P. R. China

[2]School of Materials and Energy, University of Electronic Science and Technology of China, Chengdu, Sichuan 611731, P. R. China

[3]Joint Key Laboratory of the Ministry of Education, Institute of Applied Physics and Materials Engineering, University of Macau, Avenida da Universidade, Taipa, Macau SAR, P. R. China

[4]School of Chemistry & Chemical Engineering, Anhui University of Technology, Ma'anshan, Anhui 243002, P. R. China

*Corresponding author E-mail: zengj@ustc.edu.cn (J.Z.)

[†]These authors contributed equally to this work.

Experimental Section

Chemicals and materials. Copper nitrate trihydrate ($Cu(NO_3)_2·3H_2O$, ≥99.99%), and Potassium bicarbonate ($KHCO_3$, ≥99.99%) were purchased from Aladdin Chemistry Co.. Oxalic acid ($H_2C_2O_4$, anhydrous, 99.0%), and Cerium (III) nitrate hexahydrate ($Ce(NO_3)_3·6H_2O$, 99.999%) were purchased from Sigma-Aldrich. Potassium hydroxide (KOH, 95%), and Potassium thiocyanate (KSCN, 99.5%) were purchased from Macklin. Deuterium oxide (D_2O, 99 atom% D), Dimethyl sulfoxide (DMSO, AR), Ethanol (EtOH, AR) were purchased from Sinopharm Chemical Reagent Co. Ltd (Shanghai, China). All chemicals were used as received without any further purification. Deionized water obtained from a Milli-Q water purification system (18.2 MΩ cm) was used to prepare aqueous solutions. Sustainion X37-50 Grade 60 anion exchange membrane was purchased from Dioxide Materials.

Synthesis of CuO catalyst. 4.82 g of $Cu(NO_3)_2·3H_2O$ was dissolved in 100 ml of ethanol. Subsequently, 50 ml of 0.41 mol L^{-1} oxalic acid solution (dissolved in ethanol) was pumped into the mixture at a rate of 3 ml min^{-1}. After stirring at 550 rpm for 30 minutes, the product was centrifuged, washed three times with ethanol, and dried under vacuum overnight. The obtained copper oxalate powder was evenly spread in a ceramic crucible and calcined at 623 K for 2 h with a heating ramp of 3 K min^{-1}. The resulting CuO powder was redispersed in ethanol and ultrasonicated for 30 minutes. Following 5 minutes of sedimentation, the supernatant was centrifuged and dried to obtain 20-50 nm CuO catalyst.

Synthesis of CeO_x/CuO catalyst. 500 mg of as-synthesized CuO catalyst was dispersed in 60 mL of deionized water. The mixture was stirred at 550 rpm under argon protection for 10 min. Subsequently, 104 mg of $Ce(NO_3)_3·6H_2O$ was added to the suspension. After 1 min of continuous stirring, 700 μL of 1.0 mol L^{-1} KOH solution was introduced into the system. The resulting mixture was filtered after 3 min of reaction, followed by vacuum drying. The obtained solid was calcined at 623 K for 2 h with a heating ramp of 3 K min^{-1}.

Preparation of electrodes. The as-synthesized Cu-based catalyst was dispersed in ethanol and ultrasonicated to form a homogeneous ink with a concentration of 10 mg mL^{-1}. This ink was uniformly sprayed onto carbon paper (YLS-30T used throughout this work) using a pneumatic spray gun to prepare Cu-based gas diffusion electrodes (GDEs). The catalyst loadings were controlled around 0.4 mg cm^{-2} for flow cell and MEA tests, while a higher loading of 1.5 mg cm^{-2} was employed for XAFS characterizations. Notably, no binder was added to the catalyst ink

formulation.

Electrocatalytic CO_2 reduction. The electrochemical testing for product distribution was conducted in a three-chamber flow cell separated by Nafion 117 membranes. Current or potential was controlled using a Bio-Logic (VSP-3e) electrochemical workstations. Gaseous products were analyzed via gas chromatography (GC, Clarus 690, PerkinElmer, equipped with TCD and FID detectors), while liquid-phase products were characterized by ion chromatography (ICS-600, Thermo Scientific) and ^{1}H nuclear magnetic resonance spectroscopy (400 MHz NMR). Cu-based GDEs served as the working electrode with an effective geometric area of 0.5 cm wide by 1.5 cm long. The catholyte consisted of 0.5 M $KHCO_3$ flowing at 1 mL min^{-1}. Ni foam was used as the counter electrode. The anolyte was 1 M KOH circulated at 20 mL min^{-1}. An Ag/AgCl electrode was employed as reference electrode. All potentials were converted to the reversible hydrogen electrode (RHE) scale using $E_{\mathrm{RHE}} = E_{\mathrm{Ag/AgCl}} + 0.197\ \mathrm{V} + 0.0591\ \mathrm{pH} - iR_s$. Solution resistance ($R_s$) was determined by electrochemical impedance spectroscopy (EIS) and manually compensated during testing.

The stability evaluation of CeO_x/CuO catalyst was performed in an MEA configuration using an anion exchange membrane (AEM) to separate the anode and cathode compartments. IrO_x/Ti mesh was employed as the anode, with an analyte of 0.1 M $KHCO_3$ recirculated at 20 mL min^{-1}. The cathode was supplied with humidified CO_2 at a flow rate of 50 mL min^{-1}. To collect volatile products such as ethanol and propanol from the gas phase, the outlet gas was directed into a 40-cm-high trap designed to condense and retain vapor-phase species.

The partial current density is defined as

$$j_i = \frac{x_i v n_i F p}{SRT}$$

where x_i is the concentration of a specific component quantified by GC; v is the gas flow rate; n_i is the number of electrons transferred; p is the operating pressure; F is the Faraday constant (96,485 C mol^{-1}); S is the electrode area; R is the ideal gas constant (8.314 J mol^{-1} K^{-1}); T is the operating temperature. The corresponding FE was calculated as

$$FE_i = \frac{j_i}{j_{total}} \times 100\%$$

Characterizations. TEM images were collected on Hitachi H-7650 TEM equipment, and the acceleration voltage was 100 kV. HRTEM images were collected using a FEI Titan Themis

aberration-corrected transmission electron microscope at 300 kV. HAADF-STEM images and EDS elemental mapping were carried out on a Themis Z field-emission transmission electron microscope and the accelerating voltage was 200 kV. Powder X-ray diffraction (PXRD) patterns were collected on a Philips X'Pert Pro Super diffractometer with a Cu-Kα radiation ($\lambda = 1.54178$ Å). XPS were performed on a Thermo Scientific Escalab 250Xi (X-ray source: Al K alpha radiation). XAFS spectra of the Cu K-edge were obtained at the BL11B beamline of the Shanghai Synchrotron Radiation Facility with a constant current of 200 mA, operated at 3.5 GeV under "top-up" mode. Data were recorded under fluorescence mode with a Lytle detector in a homemade *operando* H-cell. Raman spectra was collected on a LabRAM HR Evolution Raman analyzer equipped with 532-nm lasering a homemade *operando* Raman flow cell. ATR-SEIRAS was performed on a Thermo Scientific Nicolet iS50 with a resolution of 8 cm^{-1} in a homemade *operando* IR H-cell.

Computational Methods. The Vienna ab initio simulation package (VASP) was used for the spin-polarized density functional theory (DFT) calculations [1]. The Perdew-Burke-Ernzerhof (PBE) functional and projector-augmented plane wave (PAW) approach were selected for the calculations with a plane wave cut-off energy of 400 eV [2]. The strongly correlated 4f electrons was dealt with the Hubbard U (Ueff = 5.0 eV for Ce) methods [3]. A 5x5 supercell with four atomic layers was established for Cu(100) surface. The Cu_6O_6 and $Ce_3Cu_4O_9$ clusters were added to model the CuO_x/Cu interface and $CeCuO_x$/Cu interface, respectively. A ($3\times3\times1$) k-point mesh was adopted to sample the Brillouin zone and a vacuum slab of 15 Å was chosen in the z-direction to avoid the interaction between each slab. The convergence criterium for geometric relaxation was set to 0.02 eV/Å. The Gibbs free energy change is defined as $\Delta G = \Delta E + \Delta ZPE - T\Delta S$, with ΔE obtained from DFT calculations, ΔZPE indicating the zero-point energy corrections, and ΔS the change of entropy. Here the entropy of adsorbed species was considered as 0. For the elementary step of CO-CO coupling, the initial states and final states are firstly optimized. Then the transition states are searched using the climbing image nudged elastic band method (CI-NEB) [4]. The accuracy was confirmed by the vibrational frequencies analysis.

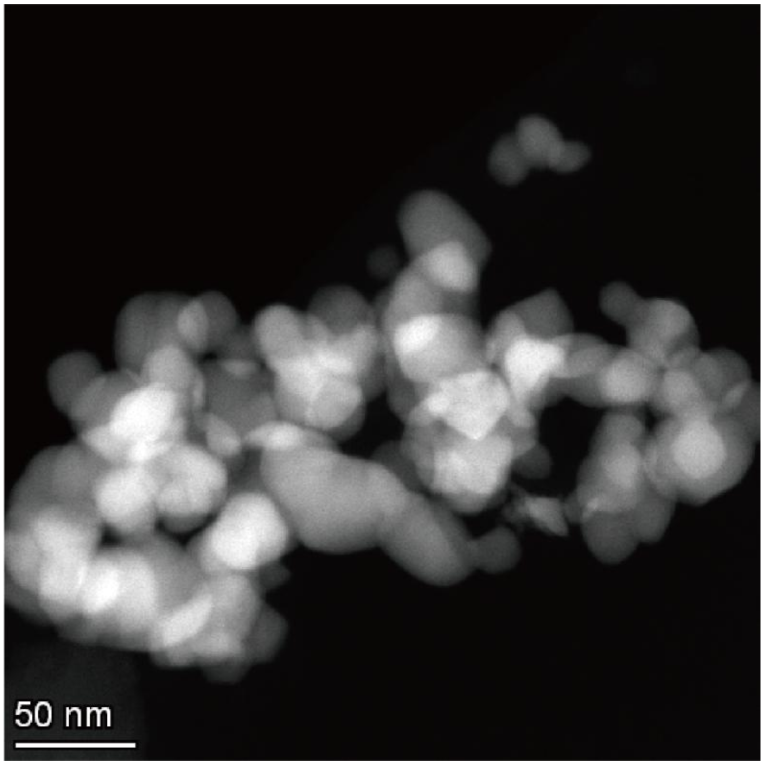

Figure S1 | HAADF-STEM of CuO nanoparticles.

Table S1 | ICP-OES of CeO_x/CuO and low- CeO_x/CuO.

	Cu 327.393 (mg L^{-1})	Ce 413.764 (mg L^{-1})	Ce/Cu mass ratio
CeO_x/CuO	771.692	16.336	0.0211
low- CeO_x/CuO	758.164	9.990	0.0132

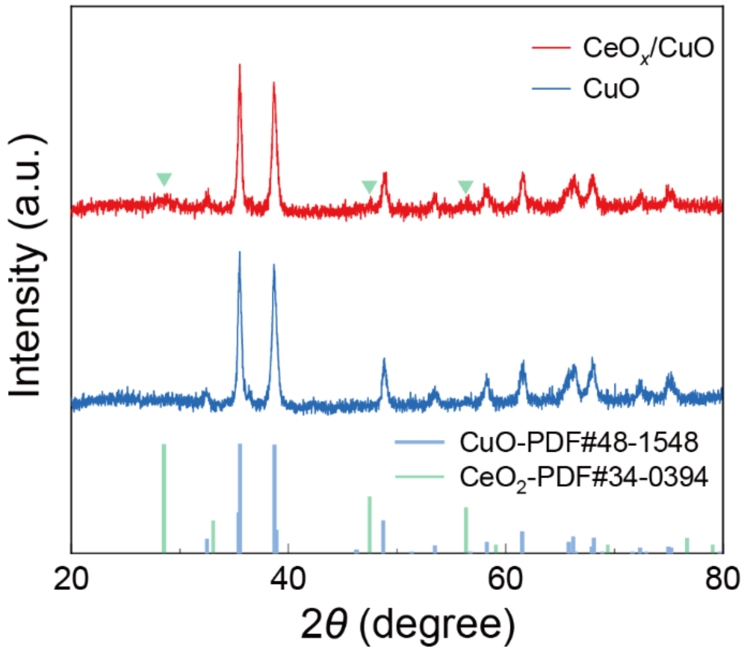

Figure S2 | XRD patterns of CeO_x/CuO (red) and CuO (blue). CeO_2 signals in CeO_x/CuO were marked in green triangles.

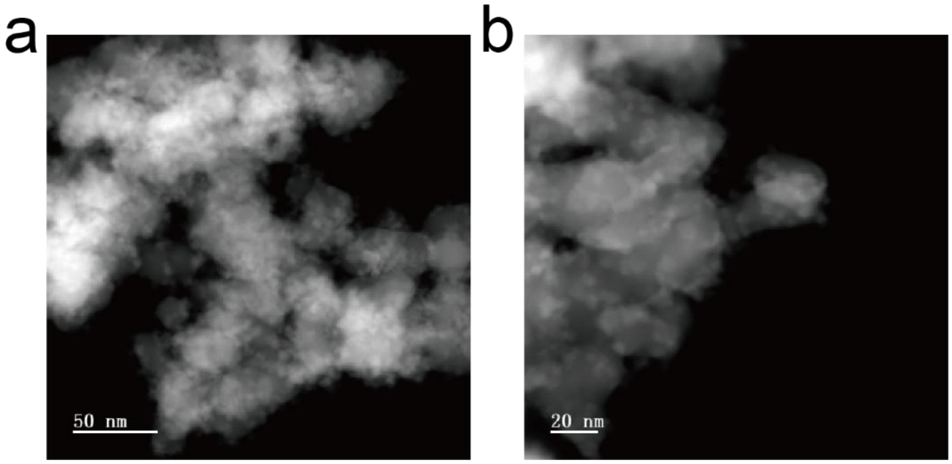

Figure S3 | a and **b** are DF-TEM images of CeO_x/CuO obtained from different fields of view.

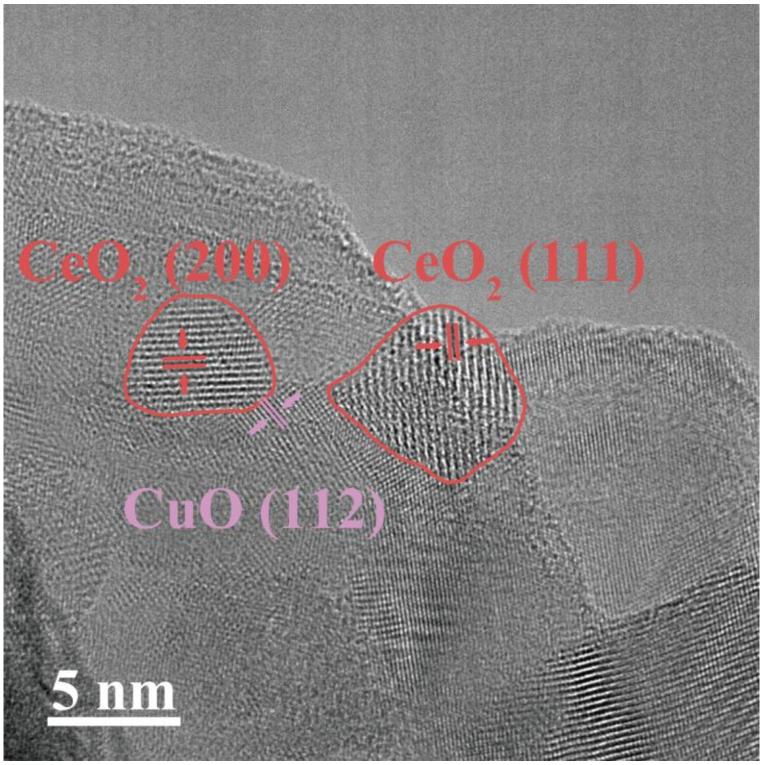

Figure S4 | HRTEM image of CeO_x/CuO. Two CeO_2 particles (in red circles) located on CuO.

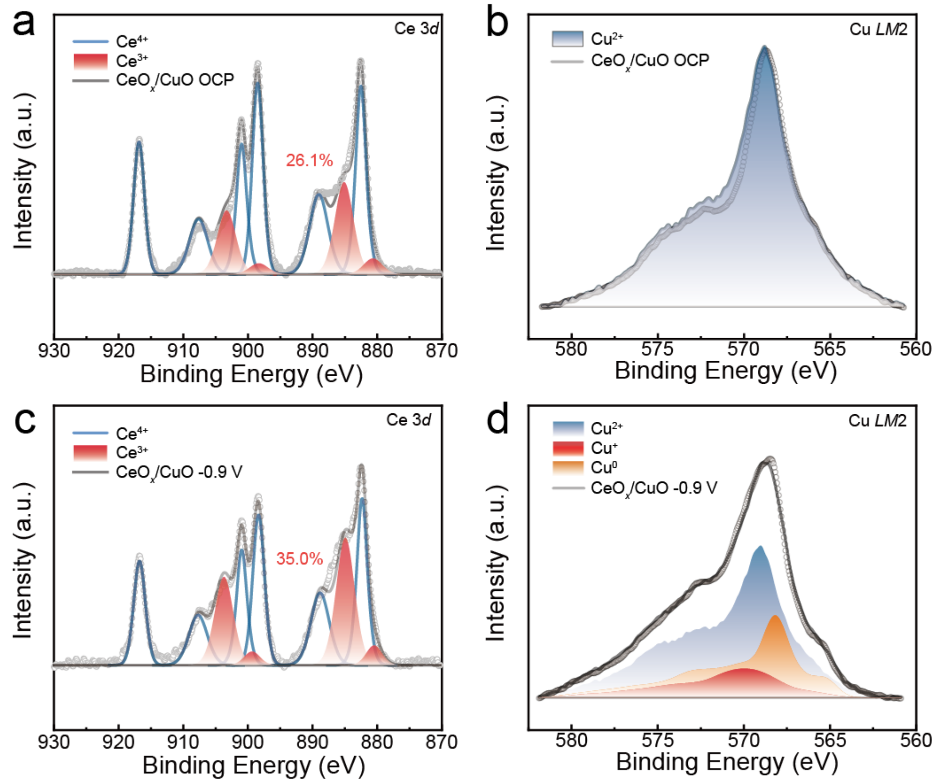

Figure S5 | Ce 3*d* XPS spectra (**a**) and Cu *LM*2 Auger spectra (**b**) of CeO_x/CuO before catalysis. *Quasi-in-situ* Ce 3*d* XPS spectra (**c**) and Cu *LM*2 Auger spectra (**d**) of CeO_x/CuO after duration test.

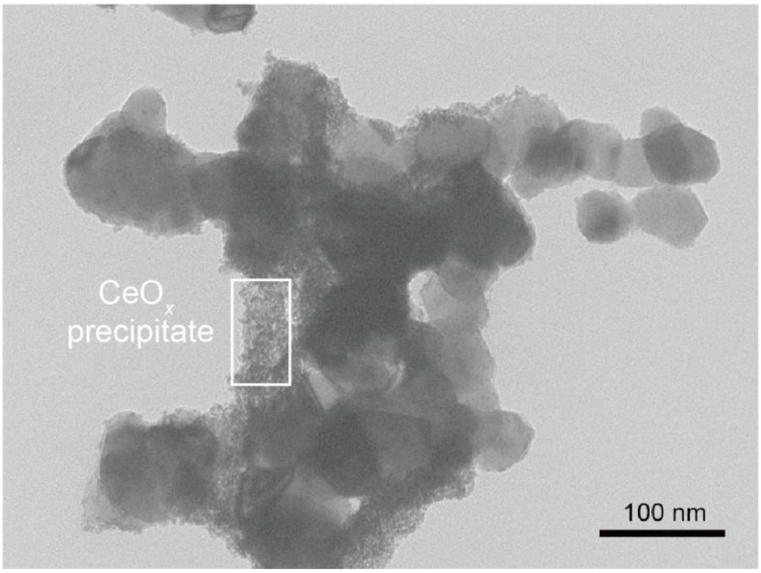

Figure S6 | TEM image of 4% Ce on CuO.

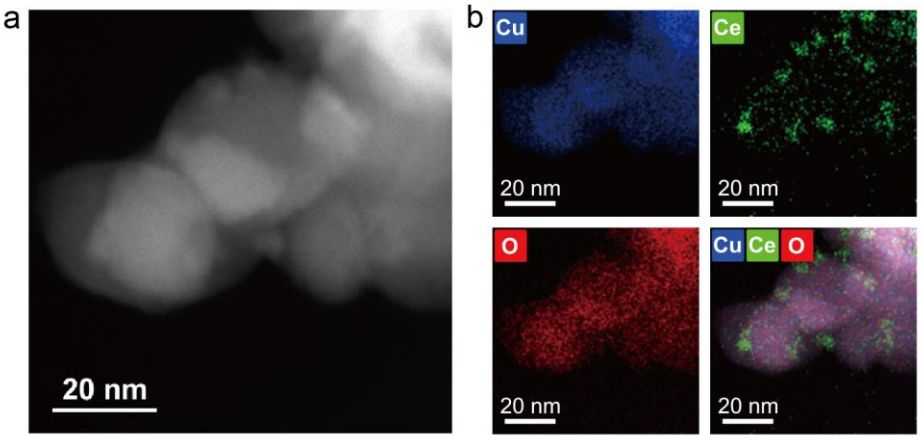

Figure S7 | a, HAADF-STEM image of low-CeO_x/CuO. **b**, The corresponding EDS mappings.

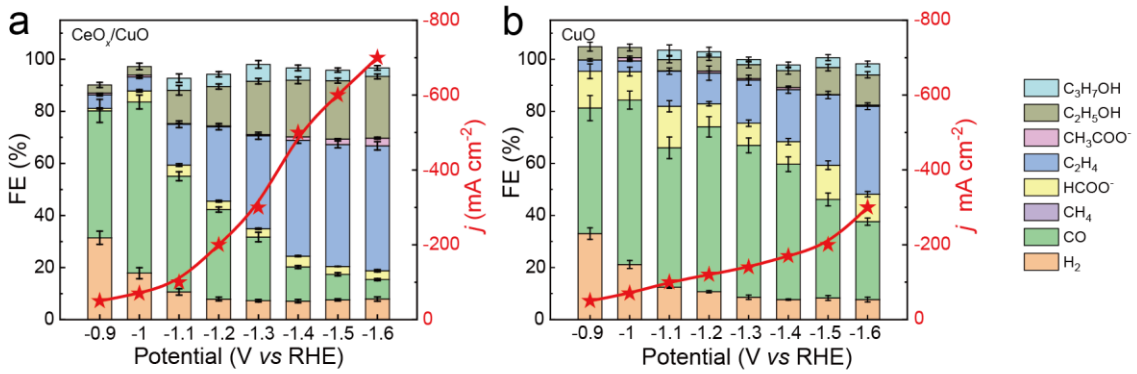

Figure S8 | Distribution of products in CO_2RR and total current densities at different potentials in flow cell of (**a**) CeO_x/CuO, and (**b**) CuO, respectively.

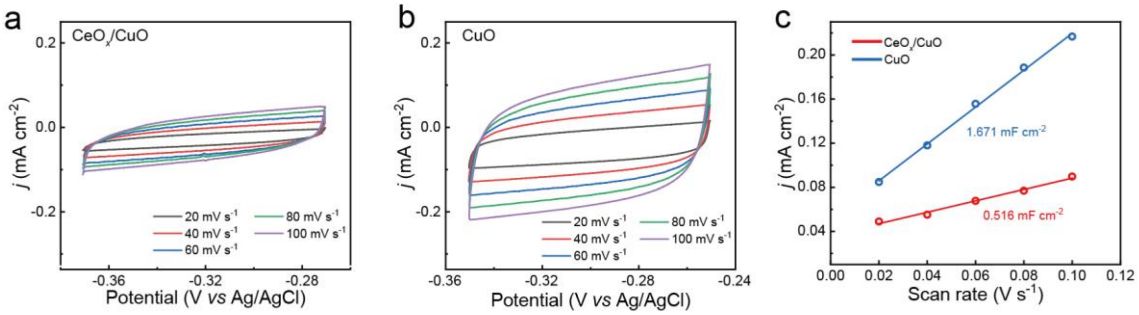

Figure S9 | CVs of (**a**) CeO_x/CuO and (**b**) CuO for double-layer capacitance measurements. (**c**) Evaluation of the double layer capacitance for CeO_x/CuO and CuO.

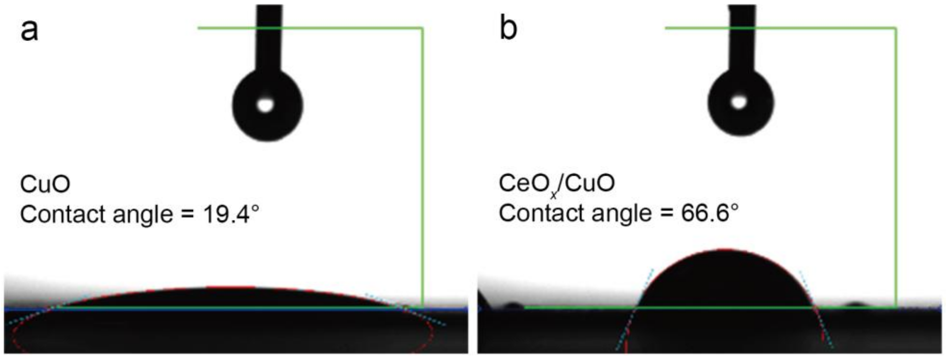

Figure S10 | The water contact angles of (**a**) CuO and (**b**) CeO_x/CuO.

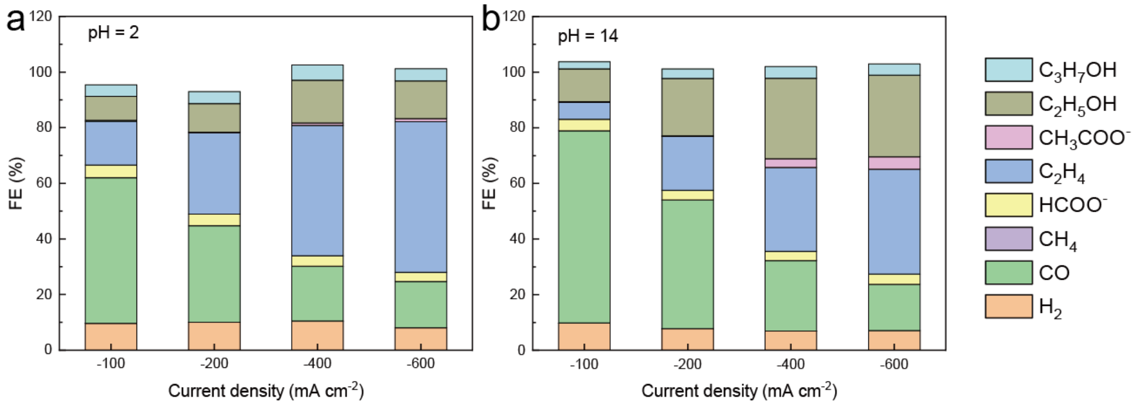

Figure S11 | Product FE of CeO_x/CuO catalyst under different pH. **a**, pH = 2 (H_2SO_4 + 0.5 M K_2SO_4). **b**, pH = 14 (1 M KOH).

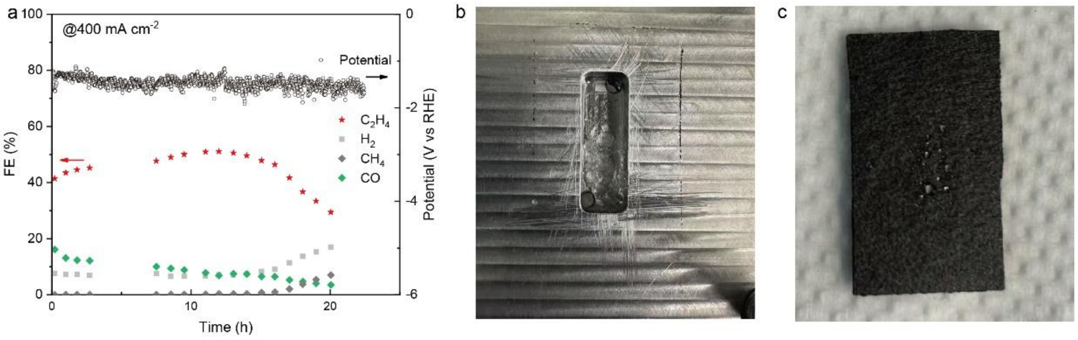

Figure S12 | a, duration test in a flow cell configuration at −400 mA cm^{-2}. **b**, the salt precipitate in gas chamber. **c**, the salt precipitate on the rear side of GDE.

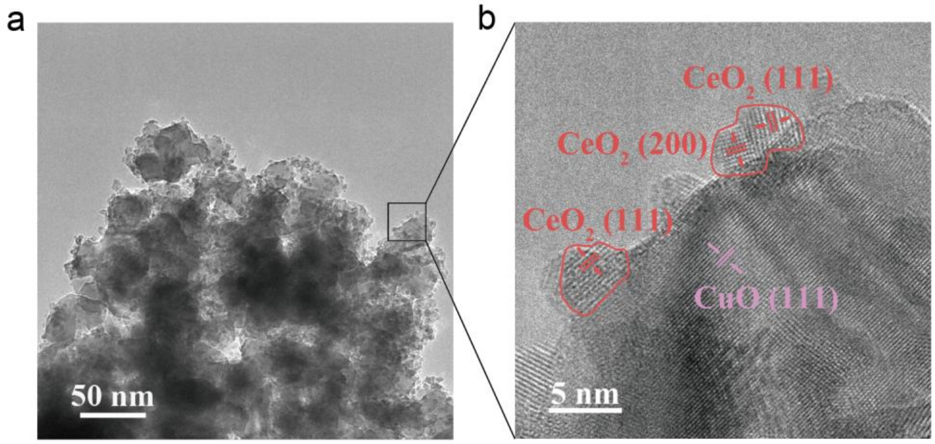

Figure S13 | a, HRTEM image of CeO_x/CuO after duration test. **b**, Enlarged view of the framed region in **a**.

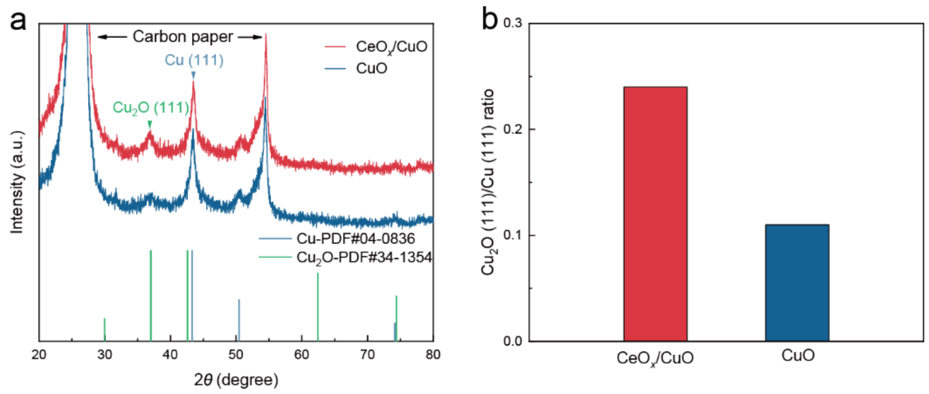

Figure S14 | XRD patterns of CeO_x/CuO (red) and CuO (blue) after reaction. The measurements were conducted after exposure to air, which likely caused partial oxidation of surface Cu species. Nonetheless, the observed phase composition supports our conclusion that CeO_x facilitates the stabilization of Cu^+ species under reaction conditions.

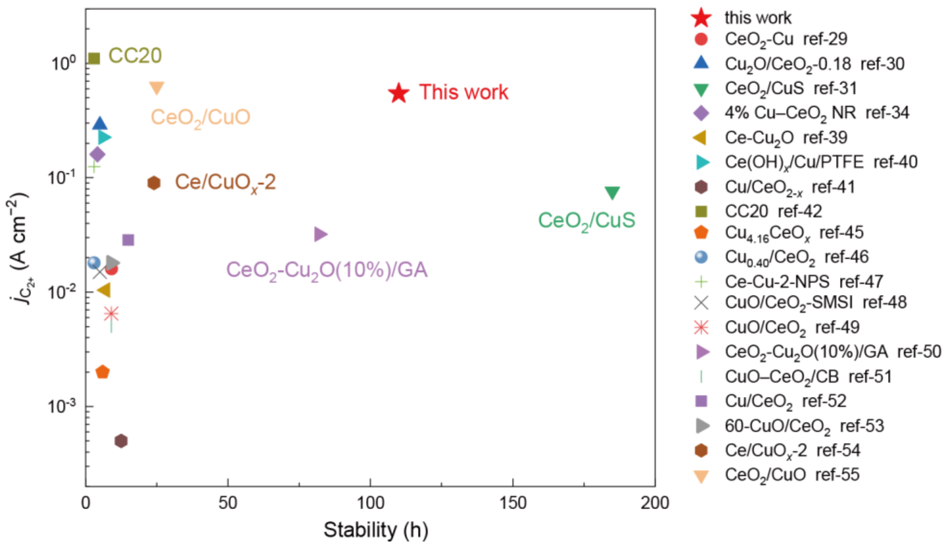

Figure S15 | Comparison of FE for C_{2+} products and current density among CuCe-based catalysts from literature and this work, with all references listed.

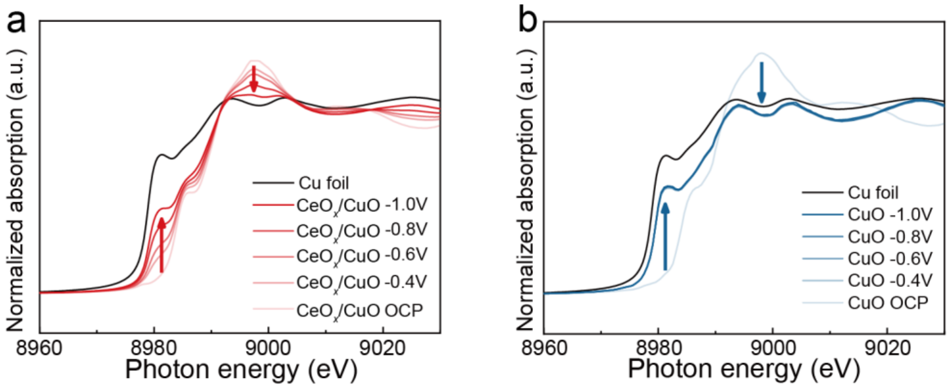

Figure S16 | Potential dependence of *operando* Cu K edge XANES spectra of (**a**) CeO_x/CuO and (**b**) CuO.

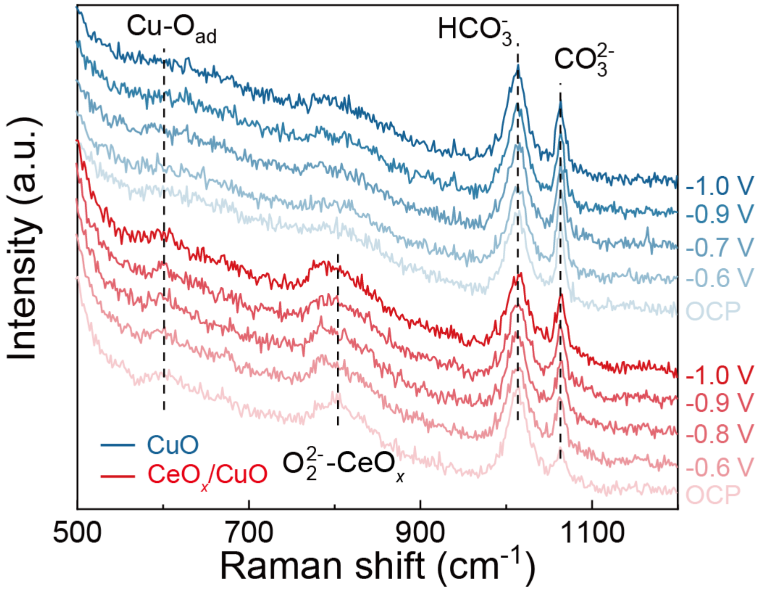

Figure S17 | *Operando* Raman spectra of CeO$_x$/CuO and CuO at different potentials. Peroxide might come from mild leakage of air.

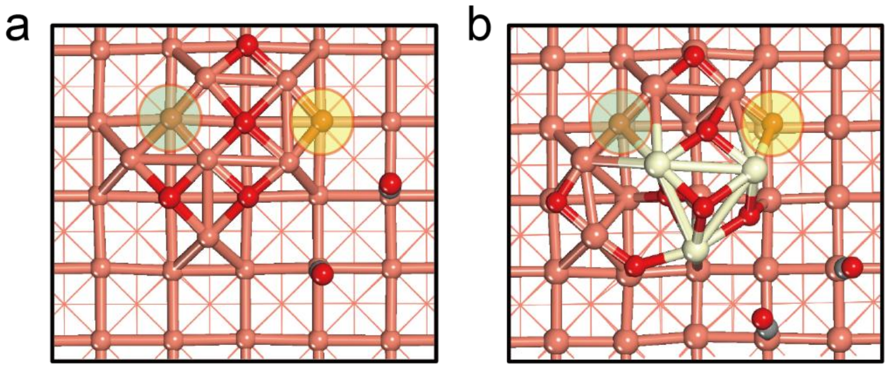

Figure S18 | Calculated O vacancy formation energies of (**a**) CuO_x and (**b**) $CeCuO_x$ on Cu (100). Oxygen coordinated by three metal atoms is marking in yellow circles, and oxygen coordinated by four metal atoms is marking in green circles. O vacancy formation energies were calculated as follow: $O_{Cu}+2H^{+}+2e^{-}$ = vac+ H_2O

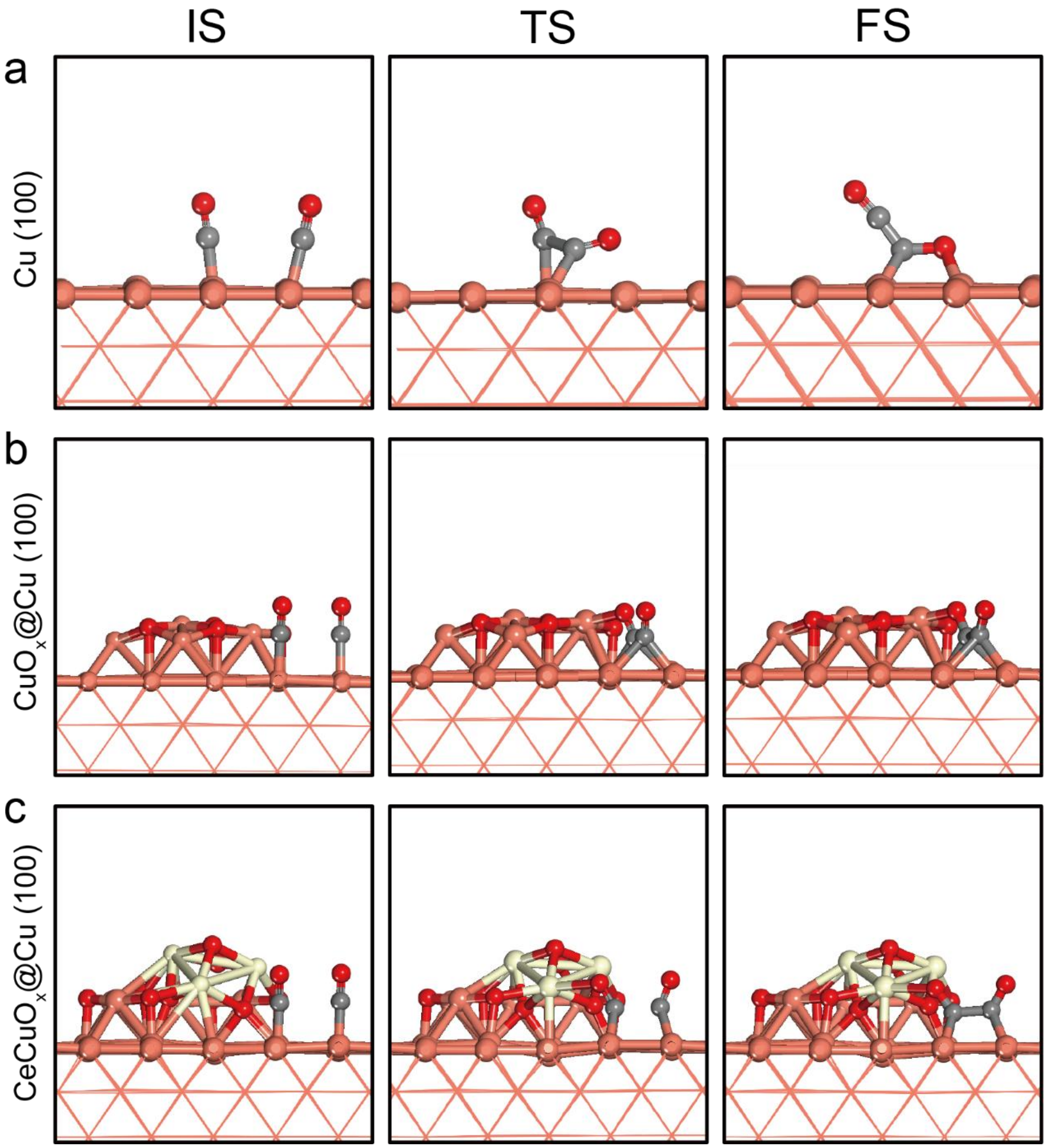

Figure S19 | Initial states (IS), transition states (TS), and final states (FS) of *CO dimerization on (**a**) Cu (100), (**b**) CuO_x@Cu (100), and (**c**) $CeCuO_x$@Cu (100).

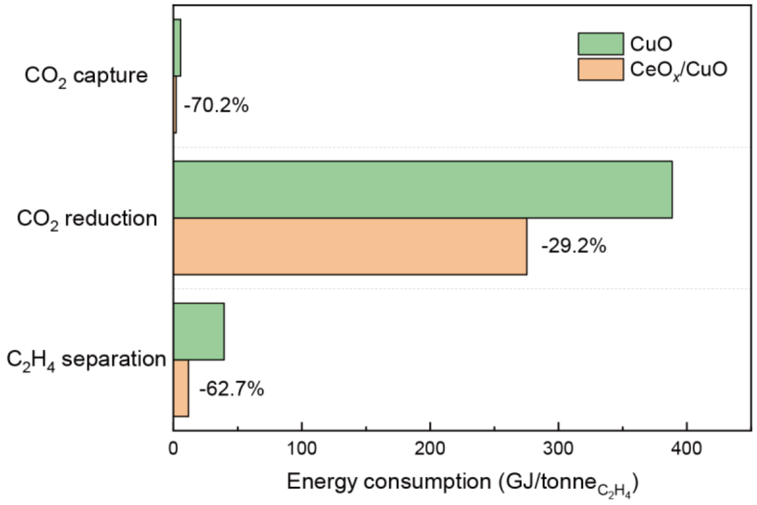

Figure S20 | The techno-economic merits of CeO_x/CuO and CuO in aspects of CO_2 capture, reduction and C_2H_4 separation.

Supplementary Note 1 | CO_2 is assumed to be captured using amine-based process with an energy cost of 4 GJ per tonne CO_2. Since the main product is C_2H_4, we hereby valuing the techno-economic merits of CeO_x/CuO in converting CO_2 to C_2H_4. The calculation of the lowest energy cost of producing one tonne of C_2H_4 is based on metrics including a H_2 FE of 8%, a CO FE of 7%, a C_2H_4 FE of 48% and a full-cell voltage of 3.2 V for CO_2 conversion to C_2H_4. For downstream gas separation, we adopted an energy consumption of 0.25 kWh/m^3 for a pressure swing adsorption (PSA) separation process. H_2, CO, and C_2H_4 are all treated as ideal gas.

Total electron transferred per tonne C_2H_4:

$$Total\ electron = \frac{Electron_{C_2H_4}}{FE_{C_2H_4}}$$

$$= \frac{Mass_{C_2H_4} \times electron\ transfer\ number \times Faraday's\ constat}{Molecular\ weight_{C_2H_4} \times FE_{C_2H_4}}$$

$$= \frac{1{,}000{,}000 \times 12 \times 96485}{28.05 \times 0.48}\ C = 8.60 \times 10^{10} C$$

Energy for CO_2 reduction:

$$Energy_{CO_2\ reduction} = Total\ electron \times Cell\ voltage$$
$$= 8.60 \times 10^{10} \times 3.2\ J = 275.29\ GJ$$

Volume of one tonne C_2H_4 at 1 bar and 273.15 K:

$$V_{C_2H_4} = \frac{Mass_{C_2H_4}}{Molecular\ weight_{C_2H_4}} \times \frac{R \times T}{p}$$

$$= \frac{1{,}000{,}000}{28.05} \times \frac{8.314 \times 273.15}{101325}\ m^3 = 799.31\ m^3$$

Volume of H_2 at 1 bar and 273.15 K when one tonne C_2H_4 is produced:

$$V_{H_2} = V_{C_2H_4} \times \frac{FE_{H_2} \times 12}{FE_{C_2H_4} \times 2}$$

$$= 799.31 \times \frac{0.08 \times 12}{0.48 \times 2}\ m^3 = 799.31\ m^3$$

Volume of CO at 1 bar and 273.15 K when one tonne C_2H_4 is produced:

$$V_{CO} = V_{C_2H_4} \times \frac{FE_{CO} \times 12}{FE_{C_2H_4} \times 2}$$

$$= 799.31 \times \frac{0.07 \times 12}{0.48 \times 2}\ m^3 = 699.40\ m^3$$

Energy for downstream gas separation:

$$Energy_{gas\ separation} = Energy_{per\ m^3} \times Total\ Volume$$

$$= Energy_{per\ m^3} \times \left(V_{C_2H_4} + V_{H_2} + V_{CO}\right)$$

$$= 0.25 \times (799.31 + 799.31 + 699.40)\ kWh = 2.07\ GJ$$

CO_2 consumption:

$$Mass_{CO_2} = \frac{Mass_{C_2H_4} \times Molecular\ weight_{CO_2}}{Molecular\ weight_{C_2H_4}} + \frac{p \times V_{CO} \times Molecular\ weight_{CO_2}}{R \times T}$$

$$= \frac{1{,}000{,}000 \times 44.01}{28.05} + \frac{101325 \times 699.40 \times 44.01}{8.314 \times 273.15}\ g = 2.94\ tonne$$

Energy for CO_2 capture:

$$Energy_{CO_2\ capture} = Mass_{CO_2} \times Energy_{CO_2\ capture\ per\ tonne}$$

$$= 2.94 \times 4\ GJ = 11.77\ GJ$$

Total energy consumption of producing one tonne C_2H_4 from CO_2 on CeO_x/CuO catalyst:

$$Energy_{total} = Energy_{CO_2\ capture} + Energy_{CO_2\ reduction} + Energy_{gas\ separation}$$

$$= 11.77 + 275.28 + 2.07\ GJ = 289.12\ GJ$$

Similar calculations of the lowest energy cost of producing one tonne of C_2H_4 on CuO catalyst were also carried out, which was based on metrics including a H_2 FE of 8%, a CO FE of 30%, a C_2H_4 FE of 34% and a full-cell voltage of 3.2 V for CO_2 conversion to C_2H_4.

References

1. Kresse G and Furthmüller J. Efficiency of ab-initio total energy calculations for metals and semiconductors using a plane-wave basis set. *Comput. Mater. Sci.* 1996; **6**: 15−50.
2. Perdew JP, Burke K and Ernzerhof M. Generalized Gradient Approximation Made Simple. *Phys. Rev. Lett.* 1996; **77**: 3865−8.
3. Nolan M, Grigoleit S, Sayle DC *et al*. Density functional theory studies of the structure and electronic structure of pure and defective low index surfaces of ceria. *Surf. Sci.* 2005; **576**: 217−29
4. Henkelman G, Uberuaga BP and Jónsson H. A climbing image nudged elastic band method for finding saddle points and minimum energy paths. *J. Chem. Phys.* 2000; **113**: 9901−4.
